# Olfactory Communication of Sickness Cues in Respiratory Infection

**DOI:** 10.3389/fpsyg.2020.01004

**Published:** 2020-06-09

**Authors:** Georgia Sarolidou, Arnaud Tognetti, Julie Lasselin, Christina Regenbogen, Johan N. Lundström, Bruce A. Kimball, Maria Garke, Mats Lekander, John Axelsson, Mats J. Olsson

**Affiliations:** ^1^Division of Psychology, Department of Clinical Neuroscience, Karolinska Institutet, Stockholm, Sweden; ^2^Stress Research Institute, Stockholm University, Stockholm, Sweden; ^3^Research Center Jülich, Institute of Neuroscience and Medicine: Jülich Aachen Research Alliance-Institute Brain Structure Function Relationship (Institut für Neurowissenschaften und Medizin-10), Jülich, Germany; ^4^Department of Psychiatry, Psychotherapy and Psychosomatics, Rheinisch Westfälische Technische Hochschule Aachen University, Aachen, Germany; ^5^Monell Chemical Senses Center, Philadelphia, PA, United States; ^6^Department of Psychology, University of Pennsylvania, Philadelphia, PA, United States; ^7^Stockholm University Brain Imaging Centre, Stockholm, Sweden; ^8^Osher Center for Integrative Medicine, Karolinska Institutet, Stockholm, Sweden

**Keywords:** sickness detection, odor perception, respiratory infection, sickness cues, body odor

## Abstract

Animals detect sick conspecifics by way of body odor that enables the receiver to avoid potential infectious transmission. Human observational studies also indicate that different types of disease are associated with more or less aversive smells. In addition, body odors from otherwise healthy human individuals smell more aversive as a function of experimentally induced systemic inflammation. To investigate if naturally occurring immune activation also gives rise to perceivable olfactory changes, we collected body odor samples during two nights from individuals with a respiratory infection as well as when they were healthy. We hypothesized that independent raters would rate the body odor originating from sick individuals as smelling more aversive than when the same individuals were healthy. Even though body odor samples from sick individuals nominally smelled more intense, more disgusting, and less pleasant and healthy than the body odor from the same individuals when healthy, these effects were not statistically significant. Moreover, raters filled out three questionnaires, Perceived Vulnerability to Disease, Disgust Scale, and Health Anxiety, to assess potential associations between sickness-related personality traits and body odor perception. No such association was found. Since experimentally induced inflammation have made body odors more aversive in previous studies, we discuss whether this difference between studies is due to the level of sickness or to the type of trigger of the sickness response.

## Introduction

Immune activation after pathogen detection has life-saving benefits but also certain metabolic and functional costs (Sheldon and Verhulst, 1996; Shakhar and Shakhar, 2015). Being able to detect and avoid pathogens before they enter the body is, therefore, of great importance, and sensory systems such as vision and olfaction could be used to detect cues of sickness in others (Schaller, 2011). Experimental studies suggest that humans can indeed detect subtle visual cues of sickness. Specifically, it has been shown that faces of individuals made experimentally sick are less red and more pale, have more hanging eyelids and droopier corners of the mouth, and are in addition rated to express more negative emotions compared to healthy faces (Henderson et al., 2017; Axelsson et al., 2018; Sarolidou et al., 2019). The role of olfaction in sickness detection has until recently been investigated predominantly in non-human animals. These studies demonstrate that sick animals’ body odors convey health status to conspecifics, leading to social avoidance (Kiesecker et al., 1999; Kavaliers et al., 2005). Similarly, experimental studies showed that rats injected with an endotoxin (lipopolysaccharide, LPS) causing a systemic inflammation were avoided by their conspecifics more compared to saline-injected rats (Arakawa et al., 2009b; Dantzer, 2009; Boillat et al., 2015). These animal studies indicate the existence of olfactory sickness cues emanating from the sick individual that promotes sickness avoidance. Because an important role of human olfaction through selective pressures of evolution (Fumagalli et al., 2009, 2011) may be to act as a warning system that enables humans to perceive cues of potential danger (Hawkes and Doty, 2009, p. 1), it is plausible that humans can also detect olfactory cues of disease states.

Along this line of reasoning, observational studies have shown a relationship between body odor, in particular breath, sweat, urine, and blood, and different diseases, such as cholera, pneumonia, tuberculosis, and smallpox, underlying the existence of olfactory sickness detection (reviewed in Penn and Potts, 1998; Shirasu and Touhara, 2011). In addition, olfactory sickness detection has been experimentally tested using body odor samples from the axilla area from healthy participants who were made sick by receiving an intravenous injection of LPS. The body odor samples have then been presented to a new group of individuals. In an initial study, the “sick” body odors were rated as more aversive and less “healthy” compared to healthy ones (Olsson et al., 2014). A more recent experiment, using the same approach, showed that the urine of immune-activated individuals had a more aversive smell and an altered composition of volatile compounds compared to the urine of healthy individuals (Gordon et al., 2018). A fMRI study utilizing the same experimental sickness model investigated the effects of sickness on social liking of individuals that were presented as combinations of facial photographs and body odors (Regenbogen et al., 2017). Faces were less liked when sick, and exposing raters to sick body odors tended to lower their liking of the faces. Olfactory and visual sickness cues resulted in increased neural activation of odor- and face-perception networks, respectively, as well as of networks involved in multisensory integration.

Overall, the aforementioned studies support the notion that olfactory sickness cues of an experimentally induced transient inflammatory response would be perceptually detected by humans. However, whether olfactory cues of commonly occurring diseases, such as the common cold or the flu, can be detected among humans is yet to be seen.

The ability to detect sickness cues and the behavioral consequences of detection are likely to vary across individuals. In particular, higher perceived vulnerability to disease is indicated to render individuals more attentive toward objects or humans that might appear contagious (Ackerman et al., 2009; Schaller, 2011). Moreover, individuals suffering from health anxiety, a persistent fear of being or falling ill, perceive others as less healthy, and they also rate the risk of contagion as greater compared to individuals with low levels of health anxiety (Salkovskis et al., 2002; Hedman et al., 2016). In parallel, individuals that are easily disgusted tend to exhibit more behavioral avoidance of sickness connoting stimuli (Fan and Olatunji, 2013). However, it has not been tested if individual traits such as disgust sensitivity, higher perceived vulnerability to disease, or health anxiety influence olfactory sickness detection.

In the present study, we investigated whether odor cues of naturally occurring respiratory infection could be detected by humans. Respiratory infections are ubiquitous world-wide (an estimated 17.1 billion cases in 2017; Gbd.2017 Disease and Injury Incidence and Prevalence Collaborators, 2018) and infect through social contact. Perceptual detection of respiratory infection would, thus, be potentially beneficial for the individual facing risk for contagion. As has been shown previously, body odors allow humans to discriminate between sick and healthy conspecifics (Olsson et al., 2014; Gordon et al., 2018). A recent study also showed that body odors from sick individuals are less liked (Sarolidou et al., 2020), indicating that sick body odors could trigger avoidance of infectious individuals.

The first aim of the current study was to investigate whether humans can discriminate between sick and healthy body odors in a natural sickness model, i.e., from individuals suffering from a respiratory infection. Our second aim was to investigate if higher perceived vulnerability to disease, levels of health anxiety, and disgust sensitivity would moderate the perception of the sickness cues. Our hypotheses were that humans will perceive sick body odors as more aversive than healthy body odor, and that those who experience high vulnerability to disease, health anxiety, and disgust sensitivity will rate body odors as more aversive than those individuals with lower scores.

## Materials and Methods

### Stimuli

**FIGURE 1:**
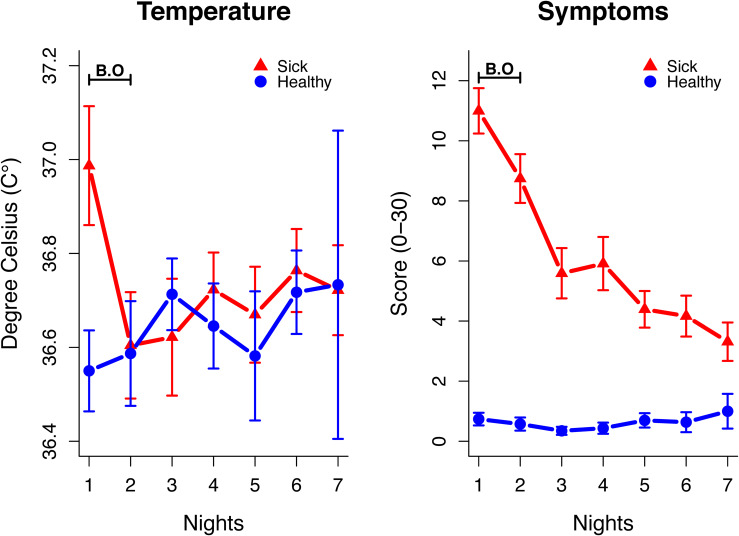
Body temperature and sickness symptoms during 7 nights for the sick and a healthy condition. Body odor collection (B.O) took place during the first two nights in each condition. Error bars represent the standard error.

The stimuli for the current behavioral experiment resulted from a naturally occurring acute respiratory infection study (Lasselin et al., 2019). Twenty-three individuals (14 women, 9 men; mean age = 32.4, *SD* = 13.3), from now on called body odor donors, donated body odors at two different sessions (sick and healthy) separated by 4 weeks. To be eligible for donation of body odors, all body odor donors had to have at least one of the following respiratory symptoms: cough, sore throat, shortness of breath, or coryza. They also had to experience one of the following systematic symptoms: fever, headache, malaise, or myalgia. Body odor donors were instructed to avoid using antipyretics or nasal sprays. Immediately after starting to experience the above symptoms, body odor donors were provided with a study kit which included a digital in-ear thermometer (Thermoscan, Braun) and questionnaires to measure their body temperature and their sickness symptoms (Figure 1). In each condition (sick and healthy), they were also provided with t-shirts with nursing pads sewn into the armpit region, which they wore for two nights. The body odor donors were instructed not to eat any strong spices, garlic, or asparagus, and to avoid alcohol, smoke, and perfumed products during the days of sampling. During the day, the t-shirts were stored in plastic bags. After completion, the study kit and the t-shirts were returned. All nursing pads were placed in glass jars and were frozen at −35°C. One body odor donor did not return a nursing pad, but only a piece of fabric from the t-shirt s/he wore and, thus, were excluded from the statistical analysis. The study was approved by the regional ethical review board in Stockholm (2011/2034-31/1). All body odor donors received 1200 SEK as a compensation for their contribution (1-week participation, repeated 1 month after first donation). For more information regarding the study protocol, and results regarding effect of sickness on sleep quality (see Lasselin et al., 2019).

### Raters

Fifty-four individuals, from now on called raters, were recruited for the present study. Due to technical problems (a failing software tool), responses from forty-six raters (34 women, 12 men; mean age = 25.2 years, *SD* = 7.0) were eligible for statistical analysis. All raters were recruited via the Karolinska Institutet online recruitment system and via posters at the Karolinska Institutet campus. To be included in the study, raters had to report to be non-smokers, not pregnant, have a normal or corrected to normal vision (required to read the scales and give their responses), a self-reported functional sense of smell, and be 18 years old or older. All raters received two cinema tickets as a compensation for their participation. The study was approved by the regional ethical review board in Stockholm (2017/55-31/4).

### Experimental Procedure

All raters were presented with the odor stimuli (23 sick and 23 healthy, described above) in a unique randomized order. Each odor pad was placed in a glass jar. Before each odor presentation, the experimenter removed the lid from the jar and presented the body odor to the rater. Before each odor presentation, a fixation cross was presented on the screen for 5 s, warning the raters for the upcoming odor presentation. The odors were presented for 3 s each. After the presentation of each body odor, the raters were presented with four visual analog scales, one at a time in fixed order, and were asked to rate the intensity, pleasantness, health, and disgust of the body odor using a computer mouse (software used: E-prime Psychology Software tools, Sharpsburg, PA, United States). Each session (fixation cross, odor presentation, rating scales) lasted 24 s. This time allowed for mitigation of any substantial habituation effect. All raters had 4 s to give ratings for each scale. The ratings were ranging from 0 to 100, where 0 indicated not intense, not pleasant, not healthy, and not disgusting at all, and 100 indicated very intense, very pleasant, very healthy, and very disgusting.

### Questionnaires

Before the experimental procedure, all raters filled out and signed an informed consent form and a demographic questionnaire, where they provided information in relation to the inclusion criteria. All raters filled in the following three questionnaires. The first questionnaire, Perceived Vulnerability to Disease (PVD), is a 15-item self-reported tool assessing individuals’ beliefs about disease transmission with two subscales: Germ Aversion and Perceived Infectability (Duncan et al., 2009). The second questionnaire, Disgust Sensitivity Revised (DS-R), is a 25-item self-reported tool. It assesses individuals’ disgust sensitivity and consists of three factors: Core Disgust, Animal Reminder Disgust, and Contamination Based Disgust (Olatunji et al., 2007, 2008). The final questionnaire, Health Anxiety Inventory (short version; HAI), is a 27-item self-reported instrument and consists of three subscales: negative consequences of illness, reassurance seeking, and avoidance behaviors (Salkovskis et al., 2002). The scores of these questionnaires were collected to be related to sickness cue perception. After the completion of the questionnaires which took place before the ratings of the body odors, the experimenter led the rater to another room for the next part of the study.

### Statistical Analyses

For the statistical analysis, two stimuli in the healthy condition were excluded due to the use of perfume, thus data from 23 body odor donors in sick condition and 21 body odor donors in healthy condition were rated by 46 raters. The effect of body odor sickness status on the perceived odor intensity, pleasantness, health, and disgust was analyzed in separate linear mixed models. For each model, the response variable was the predicted scores of one of the four scales and the fixed factor was the variable sick condition (sick vs. healthy body odors). The models specified random intercepts for raters and body odor donors. Random slopes were specified maximally, following Barr et al. (2013), but because the model did not converge, the final models used controlled for the random slope of body odor donors only.

The influence of PVD, DS-R, and HAI, on the perceived intensity, pleasantness, health, and disgust, were analyzed in separate linear mixed models. For each model, the response variable was the predicted scores in each of the four scales. Our explanatory variables were the total scores of the three questionnaires used. We also included the variable sick condition (sick vs. healthy body odors) and its interaction with each of the three questionnaires to examine whether individuals who score higher ratings in perceived vulnerability to disease, disgust sensitivity, and health anxiety will perceive sickness cues in a different way compared with individuals with lower scores in these personality traits, especially for sick body odors. We included all the interactions terms in the initial models (in the models with interaction terms, the total scores were mean centered), which were then simplified by removing all the non-significant interaction terms to achieve the minimal adequate model. We checked for multicollinearity by calculating the variance inflation factor (VIF) for all explanatory variables and the interaction terms. We ruled out potential bias from multicollinearity as all variables demonstrated a low value of VIF for all models (VIF < 1.51). The models specified random intercepts for raters and body odor donors and random slopes for body odor donors only. All analyses were conducted in R, version 3.4.2, using the package *lme4*.

## Results

### Effect of Sickness on Perceived Body Odor

Descriptive statistics of the score of Intensity, Pleasantness, Health, and Disgust are shown in Table 1.

**TABLE 1:** Descriptive statistics of the scores of Intensity, Pleasantness, Health, and Disgust rated by 46 raters.

**Scale**	**Healthy**		**Sick**	
	**Mean**	**SE**	**Mean**	**SE**
Intensity	24.46	1.36	27.25	1.84
Pleasantness	48.76	0.76	48.11	0.93
Health	49.74	0.58	48.52	0.77
Disgust	22.80	1.20	25.70	1.76

**FIGURE 2:**
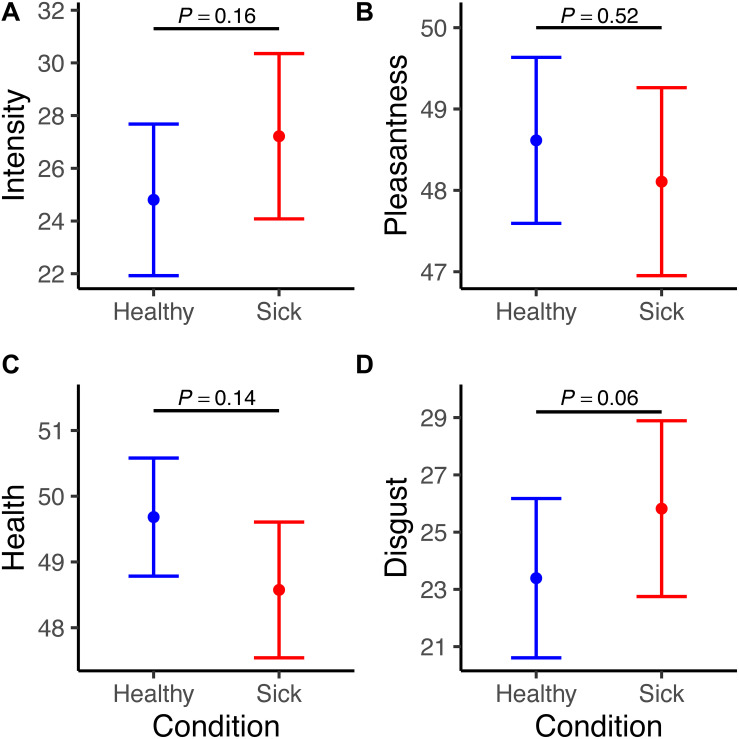
Graphs depict ratings on **(A)** intensity, **(B)** pleasantness, **(C)** health, and **(D)** disgust for both sick (red) and healthy (blue) body odors (predicted values from the Linear Mixed Models). The scales were ranging from 0 (not intense/pleasant/healthy/disgusted) to 100 (very intense/pleasant/healthy/disgusted). Error bars indicate standard error.

The linear mixed models showed that body odor of sickness did not change perceptual odor ratings. Specifically, sick individuals were rated as more intense [β = 2.42, *SE* = 1.72, χ^2^(1, *N* = 1964) = 1.99, *p* = 0.16] and disgusting [β = 2.43, *SE* = 1.27, χ^2^(1, *N* = 1927) = 3.62, *p* = 0.06], as well as less pleasant [β = -0.51, *SE* = 0.81, χ^2^(1, *N* = 1947) = 0.41, *p* = 0.52] and healthy [β = -1.11, *SE* = 0.76, χ^2^(1, *N* = 1951) = 2.14, *p* = 0.14], but the results in all cases failed to reach statistical significance (Figure 2 and Supplementary Table S1).

### Association Between Sickness-Related Traits and Perceived Body Odor

For all models, none of the interaction terms tested in the initial linear mixed models were significant (0.07 < *p* < 0.93), indicating that there was no significant interaction between sickness condition and the three questionnaires. The minimal models (interaction terms excluded) revealed no significant effect of the three questionnaires on the four different rating scales (all 0.42 < *p* < 0.93, Table 2). Models using only one questionnaire at a time show similar results (Supplementary Tables S2–S4). Similar to the results of the total scores, separate models using the subscales of each questionnaire showed no significant effect of the subscales on the rating scales (data available upon request).

**TABLE 2:** Association between sickness-related traits and body odor perception.

**Ratings**	**Predictors**	**ß**	**SE**	**χ^2^**	***df***	***P***
Intensity	Intercept	22.02	10.65			
	Sickness condition	2.31	1.72	1.81	1	0.18
	PVD score	0.05	0.24	0.04	1	0.85
	DSR score	0.10	0.18	0.31	1	0.58
	HAI score	–0.31	0.63	0.24	1	0.62
Pleasantness	Intercept	46.77	3.18			
	Sickness condition	–0.54	0.82	0.44	1	0.51
	PVD score	–0.01	0.07	0.04	1	0.85
	DSR score	0.04	0.05	0.66	1	0.42
	HAI score	0.02	0.19	0.01	1	0.91
Health	Intercept	49.10	3.10			
	Sickness condition	–1.12	0.77	2.08	1	0.15
	PVD score	–0.05	0.06	0.46	1	0.50
	DSR score	0.03	0.05	0.39	1	0.53
	HAI score	0.06	0.18	0.10	1	0.75
Disgust	Intercept	22.79	10.53			
	Sickness condition	2.47	1.29	3.68	1	0.06
	PVD score	–0.02	0.24	<0.01	1	0.93
	DSR score	0.11	0.18	0.40	1	0.53
	HAI score	–0.36	0.63	0.33	1	0.57

*Linear Mixed Models investigating the influence of perceived vulnerability to disease (PVD), disgust sensitivity (DSR), and health anxiety and infectibility (HAI) on raters’ perception (*N* = 45) of the body odors’ intensity (*N*_*obsv*_ = 1922), pleasantness (*N*_*obsv*_ = 1905), health (*N*_*obsv*_ = 1908), and disgust (*N*_*obsv*_ = 1885). For each variable, the estimate, the standard error of the mean (SE), the χ^2^ statistic, the degrees of freedom (*df*), and the *p*-value of the likelihood ratio test of the comparison between the full model and the model without the factors are given. The estimates of the variable sickness condition are for the comparison between the sick vs. healthy condition (reference category).*

## Discussion

In the present study, we investigated the ability of humans to detect sickness from olfactory cues in terms of ratings of qualities of odor samples from individuals with naturally occurring respiratory infection. Odors from sick individuals tended to smell more disgusting, more intense, and less healthy, but these ratings did not differ significantly. However, all nominal effects were in the hypothesized direction. We further investigated if individuals with more perceived vulnerability to disease, disgust sensitivity, and health anxiety perceived sickness cues as more aversive compared with individuals with lower scores in these personality traits. Neither of these hypotheses was confirmed.

As noted above, sickness detection is well established in non-human animals. Experimental studies in rodents have shown that infected animals are not only discriminated from healthy by their conspecifics, but they are also avoided more (Renault et al., 2008; Arakawa et al., 2009a). Human studies, using an experimental sickness model, have also shown that sick body odors are perceived as more aversive compared to the healthy body odors (Olsson et al., 2014; Gordon et al., 2018). This pattern could not be confirmed in the present study. Importantly, the absence of statistically significant difference in perception does not preclude other type of effects. For instance, it has been shown that differences between body odors are not necessarily observed at the level of conscious perception (Lundström and Olsson, 2005; de Groot et al., 2012) but can be seen in physiological measures such as electromyographical recordings of the facial muscles relating to different emotions.

Along the same lines, it has been suggested that odor stress cues might not be perceptually differentiated from non-stress cues. In particular, an fMRI study that examined whether olfactory signals of emotional stress can be dissociated from olfactory signals of physical stress revealed stronger amygdala activation in response to exposure to sweat related to emotional stress as compared to physical stress. Interestingly, the effect was not associated with odor discrimination, therefore excluding that as a reason of amygdala activation (Mujica-Parodi et al., 2009). In accordance, our group has recently demonstrated that body odors from experimentally immune activated individuals result in neural activation of odor specific brain networks and decreased liking of faces without any statistically significant difference between the perception of sick and healthy body odors (Regenbogen et al., 2017). It can therefore not be concluded that the lack of effects in terms of ratings translate to a lack of effect in terms of behavior, such as avoidance. However, this as well as possible main effects of naturally occurring sickness on ratings should be examined in future studies with higher statistical power.

A reason for the difference in effect of sickness on odor ratings in the present study as compared to previous LPS studies (Olsson et al., 2014; Gordon et al., 2018) may be the transient but relatively strong sickness response resulting from LPS injection. Indeed, individuals in these LPS studies reported more severe symptoms compared to the individuals who donated body odors in the present study (Lasselin et al., 2017, 2019). Specifically, headache, more intense sickness symptoms, and a higher degree of fever were present in the experimentally immune-activated individuals (Lasselin et al., 2017). Although respiratory infections lead to an inflammatory response (Newton et al., 2016), individuals who donated body odors in the present study did not experience severe sickness symptoms. Particularly, none of the body odor donors in the present study had high fever or high sickness scores in the self-reported health questionnaires during the body odor sampling period (Figure 1). Taken together, it can be speculated that the strength of olfactory sickness cues may be dependent on the presence of fever and the degree of sickness symptoms, possibly explaining the lack of significant effects in the present study.

As suggested during the review process, the existence of sex differences on perception could influence how men and women perceive sick and healthy body odors in the present study. Indeed, potential sex differences in olfaction have been assessed long ago in the literature and the results favor women. Specifically, it has been shown that women are better at detecting, identifying, and discriminating odors (Toulouse and Vaschide, 1899). Additionally, studies that specifically investigated intersexual differences in olfaction also underline women’s superiority in olfaction (Cain, 1982; Doty et al., 1985). A recent meta-analysis with a sample of 8,848 individuals showed, again, that women outperform men in all aspects of olfactory abilities (detection, identification, and discrimination; Sorokowski et al., 2019). Unfortunately, the influence of sex on the perception of sick and healthy body odors could not be performed here as our group of raters were mostly women (34 out of 46). A *post hoc* analysis on female raters showed that women perceived the sick body odors as more intense and disgusting, and less pleasant and healthy, but only disgust reached significance (*p* = 0.03) (Supplementary Figure S1 and Supplementary Table S5).

The current study also investigated how certain sickness-related personality traits suspected to influence perception of sickness influenced ratings of the olfactory stimuli. We did not find that perceived vulnerability to disease was related to the raters’ judgment of body odors in terms of intensity, pleasantness, health, and disgust. Several studies have shown that individuals who score high in the PVD questionnaire are more prone to avoid objects, humans, and actions that carry an infection risk, but also non-contagious individuals who are disabled or obese (Park et al., 2007; Schaller, 2011; Schaller et al., 2015). Also, high scores in PVD have been associated with an increased report of cues of sickness when they are just implied (Miller and Maner, 2012). It should be noted that the lack of association between PVD, disgust sensitivity, and health anxiety on the one hand, and the perception of sick and healthy body odor on the other, should be viewed in light of the fact that there were no significant perceptual differences between sick and healthy body odors.

One limitation of the present study is the relatively small sample size of both body odor donors and raters. Almost 100 individuals were initially enrolled as potential body odor donors, however, in the end, only 23 of them actually reported themselves as sick and offered body odor samples during the two sampling periods. In addition, two individuals were excluded due to suspected perfume use which made their cotton pads unsuitable for the aim of the present study. Similarly, although 54 raters were recruited, only 46 provided data that could be analyzed in the current study. Another limitation of the present study is the order of the odor sampling. Sick body odors were always collected first, meaning that possible temporal effects could have confounded the results. An unbalanced design was used because at an initial launch of the study, we observed that donors simply did not call in to start the data collection when they were randomized to start with sampling during a healthy period. As a result, it can be assumed that given that respiratory diseases are somewhat seasonal, the bias could be related to seasonal effects such as activity and sunlight. Another limitation of the study is that infectious status as measured by inflammatory markers was not determined. Although the presence of antigen during a prolonged time during naturally occurring infection could be argued to have a stronger impact on the olfactory signature of a certain individual as compared to a transient peak, we could not compare levels of inflammatory cytokines in circulation in donors of the present study with levels after exposure to LPS. The lower degree of malaise indicated by donors in the present study may indicate that the effect size in odor differences between sick and healthy is restricted. To speculate, the consistent pattern in rating the sick body odors as more aversive indicates that there may be an underlying effect which is rather small. Thus, both perception of, and behavioral responses to, body odors during naturally occurring sickness, as well as individual differences, should be investigated in future larger-scale studies. Ideally, such studies should employ a balanced design and include biological measures reflecting immunological activation.

The major strength of this study is its natural setting. It was the first time that olfactory sickness detection was tested using body odor samples collected in the homes of individuals with naturally occurring respiratory infections. As noted, respiratory infections are commonly occurring and thus it is safe to assume that all raters in the present study have multiple times encountered a person with this type of infection. Hence, they may have learned that respiratory infection is not always effectively contagious or even life threatening once the infection is contracted. At the same time social interaction is important and risking a mild infection may be a rational behavior. Future studies should investigate the effect of body odors occurring from naturally occurring respiratory infection on people with compromised immune system such as HIV or cancer patients, or older people with a lifelong exposure to infections. An interesting finding along these lines is that pregnant women, especially during their first trimester when their immune system is suppressed, express a higher disgust sensitivity to foods (Fessler et al., 2005).

## Conclusion

In conclusion, although the effects were in the hypothesized direction, body odor, during a naturally occurring respiratory infection, was not rated as significantly different as compared to body odor during a healthy condition. As previous studies using experimentally induced inflammation have shown that body odors can become more aversive, future studies should attempt to decipher whether this difference is due to the level of sickness or to the type of trigger of a sickness response or to other circumstances.

## Data Availability Statement

The datasets generated for this study are available on request to the corresponding author.

## Ethics Statement

The studies involving human participants were reviewed and approved by Regionala Etikprövningsnämnden i Stockholm (2011/2034-3111). The patients/participants provided their written informed consent to participate in this study.

## Author Contributions

MO, JA, CR, and JNL developed the study concept. MO, JA, and GS designed the study. MG collected the body odors donors’ data. GS collected the raters’ data. AT analyzed the data. MO, AT, and GS drafted the manuscript. JL, CR, JNL, BK, ML, and JA provided critical revisions. All authors approved the final version of the manuscript for submission.

## Conflict of Interest

The authors declare that the research was conducted in the absence of any commercial or financial relationships that could be construed as a potential conflict of interest.
