# Supplementary material for: Olfactory Communication of Sickness Cues in Respiratory Infection
Source: Front Psychol. 2020 Jun 9;11:1004. doi: 10.3389/fpsyg.2020.01004 (PMC7296143; doi:10.3389/fpsyg.2020.01004)

Supplementary Material

**Olfactory Communication of Sickness Cues in Respiratory Infection**

Georgia Sarolidou^1^, Arnaud Tognetti^1^, Julie Lasselin^1,2^, Christina Regenbogen^4,5^, Johan N. Lundström^1,6,7,8^, Bruce Kimball^6^, Maria Garke^1^, Mats Lekander^1,2,3^, John Axelsson^1,2,3^, Mats J. Olsson^1^

**Table S1. Effects of sickness on body odor perception.** Linear Mixed Models investigating the influence of sickness on body odors’ ratings (Ndonor=23, Nrater=46) in intensity (Nobsv=1964), pleasantness (Nobsv =1947), health (Nobsv =1951) and disgust (Nobsv =1927). For each variable, the estimate, the standard error of the mean (SE), the χ2 statistic, the degrees of freedom (df), and the *p*-value of the likelihood ratio test of the comparison between the full model and the model without the variable *sickness condition* are given. The estimates of the variable *sickness condition* are for the comparison between the sick vs. healthy condition (reference category).

| Ratings | Predictors | ß | SE | χ2 | df | *P* |
| --- | --- | --- | --- | --- | --- | --- |
|  |  |  |  |  |  |  |
| Intensity | Intercept | 24.80 | 2.88 |  |  |  |
|  | Sickness condition | 2.42 | 1.72 | 1.99 | 1 | 0.16 |
|  |  |  |  |  |  |  |
| Pleasantness | Intercept | 48.62 | 1.02 |  |  |  |
|  | Sickness condition | -0.51 | 0.81 | 0.41 | 1 | 0.52 |
|  |  |  |  |  |  |  |
| Health | Intercept | 49.68 | 0.90 |  |  |  |
|  | Sickness condition | -1.11 | 0.76 | 2.14 | 1 | 0.14 |
|  |  |  |  |  |  |  |
| Disgust | Intercept | 23.39 | 2.78 |  |  |  |
|  | Sickness condition | 2.43 | 1.27 | 3.62 | 1 | 0.06 |

**Table S2. Linear Mixed Models investigating the influence of perceived vulnerability to disease (PVD) on raters’ perception (N=45) of the body odours’ intensity (N_obsv_=1922), pleasantness (N_obsv_ =1905), health (N_obsv_ =1908) and disgust (N_obsv_ =1885).** For each variable, the estimate, the standard error of the mean (SE), the χ2 statistic, the degrees of freedom (df), and the *p*-value of the likelihood ratio test of the comparison between the full model and the model without the factors are given. The estimates of the variable *sickness condition* are for the comparison between the sick vs. healthy condition (reference category).

| Ratings | Predictors | ß | SE | χ2 | df | *P* |
| --- | --- | --- | --- | --- | --- | --- |
|  |  |  |  |  |  |  |
| Intensity | Intercept | 23.20 | 7.60 |  |  |  |
|  | Sickness condition | 2.31 | 1.72 | 1.81 | 1 | 0.18 |
|  | PVD score | 0.06 | 0.21 | 0.09 | 1 | 0.77 |
|  |  |  |  |  |  |  |
| Pleasantness | Intercept | 48.27 | 2.31 |  |  |  |
|  | Sickness condition | -0.54 | 0.82 | 0.44 | 1 | 0.51 |
|  | PVD score | 0.009 | 0.06 | 0.02 | 1 | 0.88 |
|  |  |  |  |  |  |  |
| Health | Intercept | 50.52 | 2.22 |  |  |  |
|  | Sickness condition | -1.12 | 0.77 | 2.08 | 1 | 0.15 |
|  | PVD score | -0.02 | 0.06 | 0.17 | 1 | 0.68 |
|  |  |  |  |  |  |  |
| Disgust | Intercept | 24.01 | 7.51 |  |  |  |
|  | Sickness condition | 2.47 | 1.29 | 3.68 | 1 | 0.06 |
|  | PVD score | -0.005 | 0.21 | <0.001 | 1 | 0.98 |

**Table S3. Linear Mixed Models investigating the influence of disgust sensitivity (DSR) on raters’ perception (N=45) of the body odours’ intensity (N_obsv_=1922), pleasantness (N_obsv_ =1905), health (N_obsv_ =1908) and disgust (N_obsv_ =1885).** For each variable, the estimate, the standard error of the mean (SE), the χ2 statistic, the degrees of freedom (df), and the *p*-value of the likelihood ratio test of the comparison between the full model and the model without the factors are given. The estimates of the variable *sickness condition* are for the comparison between the sick vs. healthy condition (reference category).

| Ratings | Predictors | ß | SE | χ2 | df | *P* |
| --- | --- | --- | --- | --- | --- | --- |
|  |  |  |  |  |  |  |
| Intensity | Intercept | 19.94 | 8.41 |  |  |  |
|  | Sickness condition | 2.31 | 1.72 | 1.81 | 1 | 0.18 |
|  | DSR score | 0.11 | 0.16 | 0.45 | 1 | 0.50 |
|  |  |  |  |  |  |  |
| Pleasantness | Intercept | 46.70 | 2.53 |  |  |  |
|  | Sickness condition | -0.54 | 0.82 | 0.44 | 1 | 0.51 |
|  | DSR score | 0.04 | 0.05 | 0.67 | 1 | 0.41 |
|  |  |  |  |  |  |  |
| Health | Intercept | 48.76 | 2.46 |  |  |  |
|  | Sickness condition | -1.12 | 0.78 | 2.08 | 1 | 0.15 |
|  | DSR score | 0.02 | 0.05 | 0.17 | 1 | 0.68 |
|  |  |  |  |  |  |  |
| Disgust | Intercept | 18.97 | 8.32 |  |  |  |
|  | Sickness condition | 2.47 | 1.29 | 3.68 | 1 | 0.06 |
|  | DSR score | 0.10 | 0.16 | 0.39 | 1 | 0.53 |

**Table S4. Linear Mixed Models investigating the influence of health anxiety and infectibility (HAI) on raters’ perception (N=45) of the body odours’ intensity (N_obsv_=1922), pleasantness (N_obsv_ =1905), health (N_obsv_ =1908) and disgust (N_obsv_ =1885).** For each variable, the estimate, the standard error of the mean (SE), the χ2 statistic, the degrees of freedom (df), and the *p*-value of the likelihood ratio test of the comparison between the full model and the model without the factors are given. The estimates of the variable *sickness condition* are for the comparison between the sick vs. healthy condition (reference category).

| Ratings | Predictors | ß | SE | χ2 | df | *P* |
| --- | --- | --- | --- | --- | --- | --- |
|  |  |  |  |  |  |  |
| Intensity | Intercept | 27.84 | 6.73 |  |  |  |
|  | Sickness condition | 2.31 | 1.72 | 1.81 | 1 | 0.18 |
|  | HAI score | -0.25 | 0.59 | 0.18 | 1 | 0.67 |
|  |  |  |  |  |  |  |
| Pleasantness | Intercept | 48.42 | 2.07 |  |  |  |
|  | Sickness condition | -0.54 | 0.82 | 0.44 | 1 | 0.51 |
|  | HAI score | 0.02 | 0.17 | 0.009 | 1 | 0.92 |
|  |  |  |  |  |  |  |
| Health | Intercept | 49.45 | 1.98 |  |  |  |
|  | Sickness condition | -1.12 | 0.78 | 2.08 | 1 | 0.15 |
|  | HAI score | 0.02 | 0.17 | 0.02 | 1 | 0.89 |
|  |  |  |  |  |  |  |
| Disgust | Intercept | 27.49 | 6.62 |  |  |  |
|  | Sickness condition | 2.47 | 1.29 | 3.68 | 1 | 0.06 |
|  | HAI score | -0.36 | 0.58 | 0.37 | 1 | 0.54 |

**Table S5. Effects of sickness on body odor perception in female* raters.** Linear Mixed Models investigating the influence of sickness on body odors’ ratings rated by women (Ndonor=23, Nrater=34) in intensity (Nobsv=1453), pleasantness (Nobsv =1444), health (Nobsv =1443) and disgust (Nobsv =1436). For each variable, the estimate, the standard error of the mean (SE), the χ2 statistic, the degrees of freedom (df), and the *p*-value of the likelihood ratio test of the comparison between the full model and the model without the variable *sickness condition* are given. The estimates of the variable *sickness condition* are for the comparison between the sick vs. healthy condition (reference category).

| Ratings | Predictors | ß | SE | χ2 | df | *P* |
| --- | --- | --- | --- | --- | --- | --- |
|  |  |  |  |  |  |  |
| Intensity | Intercept | 25.75 | 3.34 |  |  |  |
|  | Sickness condition | 3.45 | 1.91 | 3.22 | 1 | 0.07 |
|  |  |  |  |  |  |  |
| Pleasantness | Intercept | 47.79 | 1.12 |  |  |  |
|  | Sickness condition | -0.54 | 0.93 | 0.34 | 1 | 0.56 |
|  |  |  |  |  |  |  |
| Health | Intercept | 49.33 | 1.04 |  |  |  |
|  | Sickness condition | -1.38 | 0.83 | 2.77 | 1 | 0.10 |
|  |  |  |  |  |  |  |
| Disgust | Intercept | 24.38 | 3.24 |  |  |  |
|  | Sickness condition | 3.22 | 1.46 | 4.68 | 1 | 0.03 |

* Only 12 men participated in our study, preventing us to examine the influence of sickness cues on men’s ratings and Linear Mixed Models do not show any effect of sickness on men’s ratings (*p*-value ranging from 0.55 to 0.98).

**Fig. S1.** **Graphs depict women’s ratings on intensity (A), pleasantness (B), health (C), and disgust (D) for both sick and healthy body odors** (predicted values from the Linear Mixed Models, see Table S5). The scales were ranging 248 from 0 (not intense/pleasant/healthy/disgusted) to 100 (very intense/pleasant/healthy/disgusted). Error bars indicate standard errors.


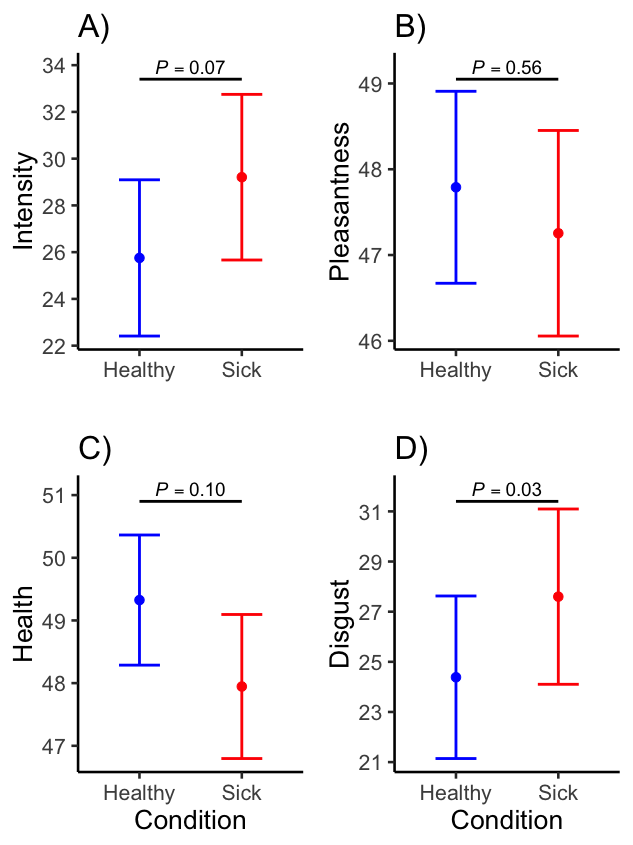

Supplement: Supplementary file 1 [file Data_Sheet_1.docx]
